# Problemas ligados ao álcool em centros de emergência (PLACE)—People experiencing homelessness with alcohol-related problems in Lisbon's emergency shelters during the COVID-19 pandemic: a description and analysis of a harm reduction intervention

**DOI:** 10.3389/fpsyg.2023.1165322

**Published:** 2023-05-19

**Authors:** Filipe Oliveira Azevedo, Ana Neto, Ana Gama, Ana Subtil, Ricardo Fuertes, Claúdia Pereira, Joana Tavares, Raquel Luis Medinas, Ana V. Silva, Sónia Dias

**Affiliations:** ^1^Department of Psychiatry and Mental Health, Centro Hospitalar de Lisboa Ocidental, Lisbon, Portugal; ^2^Unidade de Alcoologia de Lisboa, Divisão para a Intervenção em Comportamentos Aditivos e Dependências, Associação Regional de Saúde, Instituto Público, Lisbon, Portugal; ^3^Nova Escola Nacional de Saúde Pública, Public Health Research Centre, Comprehensive Health Research Center, Universidade Nova de Lisboa, Lisbon, Portugal; ^4^Câmara Municipal de Lisboa, Lisbon, Portugal; ^5^Associação Ares do Pinhal, Lisbon, Portugal

**Keywords:** homeless, alcohol, shelter, harm-reduction, COVID, pandemic, low-threshold, alcohol withdrawal syndrome

## Abstract

**Introduction:**

Alcohol-related problems disproportionally affect people experiencing homelessness. As the first wave of the COVID-2019 pandemic spread in 2020, a number of emergency shelters were opened in Lisbon. Increased difficulties in obtaining alcohol could have led to an increased incidence of alcohol withdrawal. Therefore, a low-threshold harm reduction intervention was introduced to these emergency shelters. This consisted of a fixed medication treatment, made available immediately for those with specific conditions, without the need for a medical evaluation or abstinence from alcohol, together with an offer of subsequent access to specialized addiction centers. The *Problemas Ligados ao Álcool em Centros de Emergência* (PLACE) study (alcohol-related problems in emergency shelters) is a retrospective mixed-methods observational study. It describes the demographic, health, and social characteristics of shelter users participating in the program and aims to evaluate the intervention as well as the experience of the patients, professionals, and decision-makers involved.

**Results:**

A total of 69 people using shelters self-reported alcohol-related problems. Among them, 36.2% of the people accepted a pharmacological intervention, and 23.2% selected an addiction appointment. The take-up of the intervention was associated with better housing outcomes. A description of an individual's trajectory after leaving the shelter is provided.

**Discussion:**

This study suggests that non-abstinence-focused interventions can be useful and well-tolerated in treating addiction in this population.

## 1. Introduction

Portugal is among the countries with the highest alcoholic drink consumption rates. According to the 2018 World Health Organization European Health Report, the alcohol-use disorder rate was 6.8% and alcohol dependency was 3% (World Health Organization, 2018; Teixeira, 2022). The updated version in 2021 stated that Portuguese adults tend to drink approximately 12.1 liters per year (which increased by 1.6% compared with 2015), which is higher than the average of most European countries, which is 9.5 liters per year (World Health Organization, 2022).

Alcohol-use disorder (AUD) is over-represented in people experiencing homelessness (PEH) (8.1–58.5%), although it may be underdiagnosed and undertreated (National Health Care for the Homeless Council, 2003; Fazel et al., 2008).

At the end of 2019, Lisbon was reported as having 1.071 people experiencing rooflessness and 2.883 experiencing houselessness (Grupo de Trabalho para a Monitorização e Avaliação da ENIPSSA, 2020).

In a study assessing homeless people having contact with a Lisbon psychiatric hospital from 2016 to 2019, the most common psychiatric diagnosis was drug abuse (34%), followed by alcohol abuse (33%), and numbers ranging from 41% to 77% reported in street evaluations in 1996 (Bento et al., 1996; Fernandes et al., 2022).

PEH have six to 10 times higher risk of alcohol-related death than the general population. These include not only medical complications linked to alcohol long-term abuse but also alcohol withdrawal syndrome (Hwang et al., 2009; Baggett et al., 2015).

Alcohol withdrawal syndrome (AWS) is a potentially fatal condition, occurring after sudden cessation or significant reduction in heavy and prolonged alcohol use. AWS symptoms include autonomic hyperactivity, nausea, vomiting, headache, tremors, anxiety, psychomotor agitation, and, in more severe cases, hallucinations, occupational delirium, delirium tremens, seizures, and death. It can cause irreversible neurological comorbidities, such as Wernicke–Korsakoff syndrome, which includes acute onset of Wernicke's encephalopathy (confusion, oculomotor disturbances, and ataxia), which, if untreated, can progress to or coexist with Korsakoff syndrome characterized by anterograde and retrograde memory deficits, limited learning ability, and impaired executive function (Popa et al., 2021). Additionally, Marchiafava–Bignami syndrome, a highly rare but rather severe condition characterized by demyelination and necrosis of the corpus callosum causing dementia, altered mental status, spasticity, dysarthria, ataxia, gait abnormalities, and seizures can also occur in malnourished chronic alcohol users, presumably due to combination of alcohol-induced neurotoxicity (with an uncertain nature) and deficiency of the B-complex vitamins (Singh and Wagh, 2022).

Alcohol use-related harm to PEH is aggravated by co-occurring social vulnerability, precarious health conditions, and difficulty in accessing care (Bloomfield et al., 2006; Collins et al., 2016; Stafford and Wood, 2017). Withdrawal symptoms can be a factor for a PEH to leave a welcoming center (Pauly et al., 2019). This reinforces the exclusion cycle that separates PEH from appropriate medical and social care.

During the COVID-19 pandemic, these challenges were magnified as already poor health and precarious living conditions were aggravated by reduced income, barriers to healthcare and support services, and increased vulnerability to the virus (Onyango et al., 2020).

Under the umbrella of harm reduction, safe supply prescribing and managed alcohol programs were reported as ways to mitigate potentially severe illness in emergency shelters, reduce hospital visits, and improve substance use disorder, sometimes called “risk mitigation” or “pandemic prescribing” (Bonn et al., 2020; Chang et al., 2020; Tyndall, 2020; British Columbia Centre on Substance Use (BCCSU), 2022; Brothers et al., 2022; Glegg et al., 2022).

Harm reduction interventions do not require abstinence. For alcohol, this includes a set of pragmatic strategies that minimize alcohol-related damage for the affected individual and society at large (Marlatt et al., 1998; Denning and Little, 2000).

Managed alcohol programs provide eligible individuals with regular doses of alcohol and can enhance housing stability, reduce alcohol-related harms, improve safety, and create opportunities for reconnection with families, communities, and treatment. Combined pharmacological and behavioral harm reduction regimes result in higher adherence in PEH and are effective in reducing alcohol use and associated risks as well as enhancing health and quality of life (Collins et al., 2021; Kouimtsidis et al., 2021).

Before 2020, Lisbon had no low-threshold alcohol interventions nor managed alcohol programs.

There was a considerably increased risk of AWS due to sudden reduction or suspension of alcohol consumption (due to reduced income and the closure of alcohol retailers) (Narasimha et al., 2020; Onyango et al., 2020; Rehm et al., 2020).

To prevent severe AWS, a low-threshold harm reduction intervention provided without a prior medical evaluation was made available to people experiencing homelessness during their stay at emergency shelters (ESs).

This article describes PEH with self-reported alcohol-related problems admitted to the ES from March to December 2020 and describes their participation in the harm reduction intervention. It identifies distinguishing features between those who accepted and those who rejected the intervention procedure.

## 2. Materials and methods

This is a retrospective observational cross-sectional study included in the Problemas Ligados ao Álcool em Centros de Emergência (PLACE) (alcohol-related problems in emergency shelters) research project. This article was written according to the Strengthening the Reporting of Observational Studies in Epidemiology (STROBE) Statement (von Elm et al., 2008).

### 2.1. Intervention development

The municipality of Lisbon developed four ESs, mostly adapted sports facilities, destined for PEH: *Complexo Desportivo Municipal do Casal Vistoso* (a multi-use sports structure) with a capacity for 100 individuals, *Clube Nacional de Natação* (National Swimming Club) for 48, *Pavilhão do Atlético Clube de Portugal* (a multi-use sports structure for *Atlético Clube de Portugal*) for 40, and *Casa do Lago* (a shelter created in 2020 for this purpose) for 18 (exclusive for cis and trans women) (Office of the High Commissioner for Human Rights; Fuertes et al., 2021).

These shelters provided the following:

- COVID-19 symptom triage, with daily symptom checks and temperature measurements
- Organized healthcare and social support, with a nurse on site daily and access to a physician
- In-shelter medication management
- Free meals and clothing as well as beds and showers
- Direct access to social workers and social programs
- Support for attending medical or social appointments.

While at the ES, users also had access to screenings for tuberculosis, viral hepatitis both type B and type C, syphilis, and human immunodeficiency virus (HIV), with referral and treatment when appropriate as well as access to methadone substitution programs. Different partnerships provided support with search and training for employment, documentation, language courses, and juristic support.

Substance use was not allowed inside the ES. A mobile drug consumption room was placed in front of two shelters for the use of injected substances under the supervision of health professionals. Violence, robbery, or the use of drugs and alcohol were not allowed inside the ES and were reasons for expulsion.

A low-threshold pharmacological intervention was implemented in order to reduce the incidence of severe alcohol withdrawal syndrome during the pandemic, based on a collaborative study among *Divisão de Intervenção nos Comportamentos Aditivos e Dependências* (Division for Intervention in Addictive Behaviors and Dependencies) and *Unidade de Alcoologia de Lisboa (*Alcohol Treatment Unit of Lisbon)(UAL), a center dedicated to the treatment of alcohol-related problems (ARP); the organic unit of regional health administration of Lisbon's pharmacy, the Lisbon Municipality, and the non-governmental organization *Associação Ares do Pinhal* was responsible for the clinical and social management of PEH admitted in the new ES.

The Portuguese *Serviço de Intervenção nos Comportamentos Aditivos e nas Dependências* (SICAD) (General-Directorate for Intervention on Addictive Behaviors and Dependencies) recommended that this intervention should be adopted by all ESs during the pandemic (Neto et al., 2020).

On arrival at the ES, all users were formally asked about their alcohol intake, daily use (“do you drink every day?”), and possible withdrawal symptoms (“when you don't drink, do you experience tremors, vomiting, seizures, or epilepsy?”). Clinical staff (psychologists and nurses) contributed informally to the evaluation, observing withdrawal symptoms and physical signs suggesting problematic use. No structured evaluation for alcohol-use disorder diagnosis was used as the protocol was designed to be delivered in a low-threshold principle. Those who self-reported having problematic alcohol use, informally and by answering “yes” to either question were offered a pharmacological intervention and a specialized alcohol-use appointment (at UAL or other specialized structures).

This offer was independent of any medical evaluation and did not require a prescription.

Those who accepted being engaged in the low-threshold pharmacological intervention received a fixed dose of diazepam 10 mg twice a day, tiapride 100 mg, thiamine (B1) 100 mg, pyridoxine (B6) 200 mg, folic acid (B9) 5 mg, and cobalamine (B12) 0.2 mg supplementation (Neto et al., 2020). This regular administration of medication incorporated a brief nursing intervention targeting alcohol harm reduction. This included psychological support, active listening, information about alcohol and substance use, coping strategies, participation in occupational activities, and psychosocial support.

A specialized alcohol-use medical appointment was also offered—the pharmacological intervention was delivered whether or not the individual accepted the medical appointment. The appointment took place in the ES or at UAL or at another specialized site, where users were evaluated by outreach teams that collaborated with the ES or the UAL team. Following the medical evaluation, the prescription was sometimes changed and individually tailored accordingly (e.g., some users underwent alcohol detox in the ES prior to being admitted to therapeutic communities).

The time of residence in the ES varied, but this intervention was promoted for the whole duration of residence, until drop-out, prescription of other medication, or leaving the ES.

### 2.2. Population and sample

The studied population is composed of all PEH with alcohol-related problems (ARP) that were admitted to Lisbon's Emergency Shelters from March to December 2020.

Of the 700 people housed in the ES in this time frame, 402 underwent a formal psychosocial assessment required for permanence in the shelter, and among them, 69 self-reported as having alcohol-related problems and were included in the sample.

### 2.3. Data collection

Data for the formal psychosocial assessment at admission were collected through a structured interview and included information about demographic characteristics, health and social care, and drug use. Data on the individual path/course followed within the ES was regularly registered until exitance.

The PLACE project was designed following protocol implementation and database development and was approved by the Regional Health Administration Ethical Commission (036/CES/INV/2021) (CES- *Conselho de Ética para a Saúde—*Council for Ethics in Health, INV- Investigation*)*. Anonymity and confidentiality of data were guaranteed.

### 2.4. Variables and statistical analysis

The variables under analysis regarded sociodemographic characteristics (gender, age group, marital status, education level, and country of origin), social dimensions (time homeless, family relations, social support, documentation, and ongoing judicial issues), substance use (consumption of various substances, alcohol withdrawal symptoms, overdose history, previously specialized follow-up, and risk behaviors), health status and healthcare (diagnosed physical and mental illnesses, hospital and primary care follow-up), and trajectory when leaving the shelter. Adherence to the pharmacological intervention and to the medical appointment was also reported. Other than pharmacological and alcohol-use appointment adherence data, all variables were self-reported.

The two primary outcomes were the proportion of acceptance of pharmacological intervention and the proportion of acceptance of the alcohol-use medical appointment. The secondary outcome was assessing the relationship between intervention acceptance and other variables, trying to find a pattern of acceptance moderators and facilitators.

Since all the variables under analysis were either nominal or ordinal, the descriptive analysis of the variables was carried out using absolute and relative frequencies. Continuous variables were converted to ordinal ones by grouping their values.

Pearson's chi-squared test and Fisher's exact test were applied to compare the variables under investigation between the group that adhered to pharmacological intervention and the group that did not and also between the group that adhered to the medical appointment and the group that did not.

Statistical tests were conducted at the significance level of 0.05, and a *p*-value between 0.05 and 0.1 was regarded as suggestive.

### 2.5. Confounding variables

The retrospective study design with a preexisting work database impairs controlling confounding variables at the design stage and the small *n* of this sample prevents us from doing any large-scale statistical work to minimize and elucidate their effects. We tested associations between our outcomes and all studied variables in an attempt to explore possible predictors although their meaning cannot be cleared without a logistic regression analysis which would need a considerably higher number of subjects (Hosmer et al., 2013).

However, our sample is particularly representative of the population being studied as it represents a real-world emergency scenario intervention, and outcomes and study objectives were chosen in an attempt to increase validity. Data are thus presented with this caveat which will be further approached below.

## 3. Results

Participant characteristics are presented in Table 1. Overall, 87.0% were male subjects, and 73.5% were over 40 years old. Approximately two-thirds were single (67.6%), and 22.1% were divorced. About a quarter had completed high school (12 years of education: 27.3%) and 21.2% completed 9 years (minimum mandatory schooling level), while 45.4% completed lower years of education. The majority of the participants were born in Portugal (63.9%); foreign-born participants were mostly from African countries (Angola, Cape Verde, the Gambia, Malawi, and Mozambique), and a minority were from Europe (Belgium, Moldavia, Poland, Russia, and Ukraine), South America (Brazil), and Asia (Nepal).

**Table 1:** Characteristics of the participants.

		***n* (%)**
**Sociodemographics**		
Gender	Female	9 (13.0)
(*N* = 69)	Male	60 (87.0)
	Transgender persons	0 (0.0)
Age group	27–30	3 (4.4)
(*N* = 68)	31–40	15 (22.1)
	41–50	26 (38.2)
	51–60	20 (29.4)
	61–67	4 (5.9)
Marital status	Single	46 (67.6)
(*N* = 68)	Marriage	5 (7.4)
	Divorced	15 (22.1)
	Widowed	2 (2.9)
Education	4 Years	15 (22.7)
(*N* = 66)	6 Years	15 (22.7)
	9 Years	14 (21.2)
	High school	18 (27.3)
	University	4 (6.1)
Country of origin	Portugal	39 (63.9)
(*N* = 61)	Africa	12 (19.7)
	Europe	5 (8.2)
	Latin America	3 (4.9)
	Asia	2 (3.3)
**Social dimensions**		
Time homeless	1–6 months	15 (23.1)
(*N* = 65)	6–12 months	8 (12.3)
	12–24 months	15 (23.1)
	>24 months	27 (41.5)
Maintained family relationships	Yes	27 (39.1)
(*N* = 69)	No	42 (60.9)
Social support	Yes	42 (62.7)
(*N* = 67)	No	25 (37.3)
Documentation	Yes	48 (69.6)
(*N* = 69)	No	21 (30.4)
Ongoing judicial issues	Yes	9 (13)
(*N* = 69)	No	60 (87)

More than half of the participants (58.5%) had been in a homeless situation for up to 2 years, and 41.5% for a longer period. Only 39.1% of participants reported maintaining their family relationships. The majority (62.7%) reported receiving some sort of social support (e.g., money and medication dispensing). Approximately 30% were missing some sort of documentation, either through loss (Portuguese citizens) or through bureaucratic hold-ups or lack of resources to start documentation processes (migrants), and 13% reported ongoing judicial issues, such as legal processes.

Participants were asked about health-related information (Table 2). Overall, 31.9% reported using some sort of substance recreationally other than alcohol. Among those who used substances, cannabis was the most frequently reported (50%, *n* = 11), followed by cocaine (36.4%, *n* = 8), non-prescribed sedatives (31.8%, *n* = 7), and opioids (22.7%, *n* = 5); 35% reported using more than one substance. Of the total samples, 46.4% had a history of alcohol-related withdrawal symptoms and 30.9% of overdose (of the latter, 16.1% were no longer using substances). Over half (55.1%) had previous specialized treatment achieving some period of abstinence (such as admission for detoxification or therapeutic communities). More than a third (35.4%) reported risk-taking behaviors at some point in their life (e.g., sharing needles or other materials and unprotected sex).

**Table 2:** Reported substance use, health status, and health care.

		***n* (%)**
**Substance use**		
Use of substance other than alcohol (*n* = 69)	Yes	22 (31.9)
	No	47 (68.1)
**Among those that answer Yes**		
Cannabis (*n* = 22)	Yes	11(50)
	No	11(50)
Sedatives (*n* = 22)	Yes	7(31.8)
	No	15(68.2)
Opioids (*n* = 22)	Yes	5(22.7)
	No	17(77.3)
Cocaine (*n* = 22)	Yes	8(36.4)
	No	14(63.6)
Multiple drugs (*n* = 20)	Yes	7(35)
	No	13(65)
Alcohol withdrawal symptoms (*n* = 69)	Yes	32 (46.4)
	No	37 (53.6)
Overdose history (*n* = 68)	Yes	21 (30.9)
	No	47 (69.1)
Previous follow-up at CAD (*n* = 69)	Yes	30 (43.5)
	No	39 (56.5)
Risk behaviors (*n* = 69)	Yes	23 (35.4)
	No	42 (64.6)
**Health status and health care**		
Diagnosed physical illnesses (*n* = 67)	Yes	41 (61.2)
	No	26 (38.8)
Diagnosed mental illnesses (*n* = 65)	Yes	15 (23.1)
	No	50 (76.9)
Hospital follow-up (*n* = 69)	Yes	37 (53.6)
	No	32 (46.4)
Primary Care follow-up (*n* = 69)	Yes	23 (33.3)
	No	46 (66.7)

The majority of the participants (61.2%) reported having a diagnosed physical illness (mostly non-communicable diseases such as heart disease, liver disease, diabetes, and high blood pressure), and 21.7% reported a diagnosed psychiatric disease (e.g., depression and schizophrenia). Overall, 63.8% of the participants reported being followed in a healthcare unit (non-substance use related), mostly hospital care (53.6%) and primary care (33.3%).

The participants who adhered to the pharmacological intervention did not differ significantly from those who declined it in terms of sociodemographic characteristics, social dimensions of substance use, and healthcare, except regarding health status (Table 3). A higher proportion of participants who adhered to the pharmacological intervention reported having a history of alcohol-related withdrawal symptoms (60 vs. 38.6% of those who declined; *p* < 0.10).

**Table 3:** Factors associated with adherence to the pharmacological intervention.

		**Adherence to the pharmacological intervention**				***P value***
		**Yes** ***n*** **(%)**		**No** ***n*** **(%)**		
Total		25	(36.2)	44	(63.8)	
**Sociodemographics**						
Gender	Female	3	(12.0)	6	(13.6)	*>0.999^*a*^*
	Male	22	(88.0)	38	(86.4)	
Age group	21–30	0	(0.0)	3	(7.0)	*0.387^*a*^*
	31–40	7	(28.0)	8	(18.6)	
	41–50	10	(40.0)	16	(37.2)	
	51–60	8	(32.0)	12	(27.9)	
	61 and above	0	(0.0)	4	(9.3)	
Marital status	Single	17	(68.0)	29	(67.4)	*0.575^*a*^*
	Marriage	1	(4.0)	4	(9.3)	
	Divorced	7	(28.0)	8	(18.6)	
	Widowed	0	(0.0)	2	(4.7)	
Education	4 Years	5	(20.8)	10	(23.8)	*0.531^*b*^*
	6 Years	6	(25.0)	9	(21.4)	
	9 Years	4	(16.7)	10	(23.8)	
	High School	6	(25.0)	12	(28.6)	
	University	3	(12.5)	1	(2.4)	
Country of origin	Portugal	14	(63.6)	25	(64.1)	*0.987^*a*^*
	Africa	5	(22.7)	7	(17.9)	
	Europe	1	(4.5)	4	(10.3)	
	Latin America	1	(4.5)	2	(5.1)	
	Asia	1	(4.5)	1	(2.6)	
**Social dimensions**						
Time homeless	1–6 months	5	(20.0)	10	(25.0)	*0.993^*a*^*
	6–12 months	3	(12.0)	5	(12.5)	
	12–24 months	6	(24.0)	9	(22.5)	
	3–5 years	0	(0.0)	1	(2.5)	
	5–10 years	11	(44.0)	14	(35.0)	
	Over 20 years	0	(0.0)	1	(2.5)	
Family relationships	Yes	10	(40.0)	17	(38.6)	*0.911^*b*^*
	No	15	(60.0)	27	(61.4)	
Social Support	Yes	16	(66.7)	26	(60.5)	*0.615^*b*^*
	No	8	(33.3)	17	(39.5)	
Ongoing judicial issues	Yes	1	(4.0)	8	(18.2)	*0.141^*a*^*
	No	24	(96.0)	36	(81.8)	
Documentation	Yes	20	(80.0)	28	(63.6)	*0.156^*b*^*
	No	5	(20.0)	16	(36.4)	
**Substance use**						
Use of substance other than alcohol	Yes	7	(28.0)	15	(34.1)	*0.602^*b*^*
	No	18	(72.0)	29	(65.9)	
Overdose history	Yes	6	(24.0)	15	(34.9)	*0.349^*b*^*
	No	19	(76.0)	28	(65.1)	
Previous follow-up at center for addiction disorders	Yes	9	(36.0)	21	(47.7)	*0.345^*b*^*
	No	16	(64.0)	23	(52.3)	
Risk behaviors	Yes	8	(32.0)	15	(37.5)	*0.652^*b*^*
	No	17	(68.0)	25	(62.5)	
Alcohol withdrawal symptoms	Yes	15	(60.0)	17	(38.6)	0.087^*b^
	No	10	(40.0)	27	(61.4)	
**Health care**						
Diagnosed physical illnesses	Yes	15	(60.0)	26	(61.9)	*0.877^*b*^*
	No	10	(40.0)	16	(38.1)	
Diagnosed mental illnesses	Yes	4	(16.0)	11	(27.5)	*0.284^*b*^*
	No	21	(84.0)	29	(72.5)	
Hospital follow-up	Yes	13	(52.0)	24	(54.5)	*0.839^*b*^*
	No	12	(48.0)	20	(45.5)	
Primary Care follow-up	Yes	8	(32.0)	15	(34.1)	*0.859^*b*^*
	No	17	(68.0)	29	(65.9)	
Adherence to alcohol-use medical appointment	Yes	14	(56.0)	2	(4.5)	< 0.001^***b^
	No	11	(44.0)	42	(95.5)	

^a^Fisher's exact test.

^b^Chi-square test.

Statistically significant differences at the ^*^0.1, ^**^0.05, ^***^ < 0.001 significance level.

Overall, 23.2% of the participants (*n* = 16) adhered to the alcohol-use medical appointment, and 76.8% did not attend it (*n* = 53) (Table 4). A higher proportion of participants who adhered to the medical appointment reported having maintained their family relationships (62.5 vs. 32.1% of those who did not attend, *p* < 0.05), and a lower proportion reported having a diagnosed mental illness (6.4 vs. 28.6% of those who did not attend the appointment, *p* < 0.1).

**Table 4:** Factors associated with adherence to the alcohol-use medical appointment.

**Adherence to the alcohol-use medical appointment**						***P value***
		**Yes** ***n*** **(%)**		**No** ***n*** **(%)**		
Total		16	(23.2)	53	(76.8)	
**Sociodemographics**						
Gender	Female	4	(25.0)	5	(9.4)	0.197^(1)^
	Male	12	(75.0)	48	(90.6)	
Age group	21–30		—	3	(5.8)	0.892^(1)^
	31–40	4	(25.0)	11	(21.2)	
	41–50	7	(43.8)	19	(36.5)	
	51–60	5	(31.3)	15	(28.8)	
	61 and above		—	4	(7.7)	
Marital status	Single	11	(68.8)	35	(67.3)	>0.999^(1)^
	Marriage	1	(6.3)	4	(7.7)	
	Divorced	4	(25.0)	11	(21.2)	
	Widowed	0	—	2	(3.8)	
Education	4 Years	4	(25.0)	11	(23.8)	0.603^(2)^
	6 Years	2	(12.5)	13	(21.4)	
	9 Years	4	(25.0)	10	(23.8)	
	High School	4	(25.0)	14	(28.6)	
	University	2	(12.5)	2	(2.4)	
Country of origin	Portugal	10	(71.4)	29	(61.7)	0.572^(1)^
	Africa	3	(21.4)	9	(19.1)	
	Europe		—	5	(10.6)	
	Latin America		—	3	(6.4)	
	Asia	1	(7.1)	1	(2.1)	
**Social dimensions**						
Time homeless	1–6 months	3	(18.8)	12	(24.5)	0.986^(1)^
	6–12 months	2	(12.5)	6	(12.2)	
	12–24 months	4	(25.0)	11	(22.4)	
	3–5 years		1	(2.0)		
	5–10 years	7	(43.8)	18	(36.7)	
		over 20 years	—	1	(2.0)	
Family relationships	Yes	10	(62.5)	17	(32.1)	0.029^**^(2)
	No	6	(37.5)	36	(67.9)	
Social support	Yes	11	(68.8)	31	(60.8)	0.565^(2)^
	No	5	(31.3)	20	(39.2)	
Ongoing judicial issues	Yes	1	(6.3)	8	(15.1)	^(1)^
No	15	(93.7)	45	(84.9)	*0.674*
Documentation	Yes	13	(81.3)	35	(66.0)	0.356^(2)^
	No	3	(18.7)	18	(34.0)	
**Substance use**						
Use of substance other than alcohol	Yes	5	(31.3)	17	(32.1)	0.950^(2)^
	No	11	(68.7)	36	(67.9)	
Overdose history	Yes	2	(12.5)	19	(36.5)	0.120^(2)^
No	14	(87.5)	33	(63.5)	
Previous follow-up at CAD	Yes	6	(37.5)	24	(45.3)	0.582^(2)^
No	10	(62.5)	29	(54.7)	
Risk behaviors	Yes	6	(37.5)	17	(34.7)	0.838^(2)^
No	10	(62.5)	32	(65.3)	
Alcohol withdrawal symptoms	Yes	8	(50.0)	24	(45.3)	0.740^(2)^
No	8	(50.0)	29	(54.7)	
**Health care**						
Diagnosed physical illnesses	Yes	10	(62.5)	31	(60.8)	0.902^(2)^
No	6	(37.5)	20	(39.2)	
Diagnosed mental illnesses	Yes	1	(6.3)	14	(28.6)	0.091^*^(2)
No	15	(93.7)	35	(71.4)	
Hospital follow-up	Yes	9	(56.3)	28	(52.8)	0.810^(2)^
No	7	(43.7)	25	(47.2)	
Primary Care follow-up	Yes	7	(43.7)	16	(30.2)	0.313^(2)^
No	9	(56.3)	37	(69.8)	

^(1)^Fisher's exact test.

^(2)^Chi-square test.

Statistically significant differences at the ^*^0.1, ^**^0.05, ^***^ < 0.001 significance level.

Adherence to the pharmacological intervention was significantly associated with adherence to the alcohol-use medical appointment (*p* < 0.001).

Of the 14 participants who adhered to both pharmacological intervention and medical appointment: 11 were male subjects, 10 were ≥40 years old, 9 had ≥9 years of education, 8 were born in Portugal, 10 were in a homeless situation for over 1 year, and four reported using some sort of substance recreationally other than alcohol (data not shown in Table).

Of the 42 participants, those who declined both the pharmacological intervention and the medical appointment were of the following characteristics: 37 (88.1%) were male subjects, 15 (35.7%) were 41–50 years old, 11 (26.2%) were 51–60 years old, 12 (28.6%) had high school education, 27 (64.2%) had up to 9 years, 23 (53.8%) were born in Portugal, seven (16.7%) were born in an African country, 15 (35.8%) were in a situation of homelessness for over 2 years, 10 (23.8%) were in a situation of homelessness for < 6 months, and 14 (33.3%) reported using some sort of substance recreationally other than alcohol (data not shown in Table).

Overall, 29.9% of the participants (*n* = 20) left the shelter for some sort of housing solution, and 41.8% (*n* = 28) were integrated into institutions (e.g., a shelter or a drug rehabilitation structure). The remaining 28.4% had a negative outcome–4.5% abandoned the shelter, 19.4% were expelled (reasons included using drugs in the ES, violence, or stealing) and 4.5% (*n* = 3) left due to other reasons (e.g., hospital admission, arrest, or deportation).

Of the participants who accepted the pharmacological intervention, 43.5% went on to a housing facility and 43.5% to an institution, while 13% had a negative outcome leaving the shelter. Among those who declined the pharmacological intervention, over a third (36.4%) had negative outcomes, while 40.9% went on to an institution and a minority (22.7%) followed a housing option (*p* < 0.1).

Among the participants who accepted the medical appointment, 56.3% left the shelter for a housing solution and only 6.3% had a negative trajectory outcome. Among those who refused the appointment, only 21.6% went on to a housing solution, while 43.1% were integrated into an institution and 35.3% had negative outcomes (*p* < 0.05).

## 4. Discussion

Our sample of 69 individuals constituted 17.1% of those admitted to emergency shelters with self-reported alcohol-related problems. This is much lower than the rates of 38–70% of alcohol-use disorder prevalence in homeless people that are found through formal screening instruments in foreign series and lower than the 33% to 41% in Portuguese series (Bento et al., 1996; Canadian National Health Care for the Homeless Council, 2003; Fazel et al., 2008; Fernandes et al., 2022). There are considerable challenges to self-recognizing alcohol-related problems in the general population as well as in people in a homeless situation due to potential or imagined consequences of reported consumption, social desirability, poor episodic memory, or other cognitive impairments, etc., (Grüner Nielsen et al., 2021).

One possible contributory factor is that individuals wanting to be admitted to a shelter might choose to omit information about their drinking because of a fear that this would lead them to be denied admission. Of course, drug and alcohol use were not allowed inside the shelter, but substance use was allowed outside. The sample includes only those who self-identified as having problematic alcohol use by answering the triage questions. This is consistent with a harm reduction model where patients' values and goals are prioritized, although, at the same time, it is likely to underestimate the real prevalence of alcohol-use problems. The introduction of formal screening tools can improve accuracy but would need to be modified to be consistent with harm reduction principles.

Overall, 36.2% of the identified drinkers adhered to the pharmacological intervention. In this group, there was a significant specialized medical appointment adherence of 56% among those who accepted intervention, and 23.2% of the identified sample attended an appointment. In total, two patients who refused the pharmacological intervention accepted and attended an appointment.

Outcomes may be deemed as suggestive of an association (*p*-value < 0.1) between alcohol withdrawal symptoms and pharmacological intervention acceptance, which was expected. Interestingly, maintaining family relationships was associated with adhering to a specialized appointment. This might reflect a higher social functioning of those who would engage in treatment or it may highlight the family's role in supporting alcohol-use treatment (Atadokht et al., 2015).

A significant association was found between adherence to a medical consultation in a specialist treatment unit and better housing outcomes at the end of 2020 (*p*-value < 0.05). The findings also pointed to a possible association between accepting the pharmacological intervention and a better housing trajectory, but this can only be regarded as suggestive (*p*-value < 0.1). Although we have a small sample and this is a particular crisis environment, it replicates other authors' findings that harm reduction interventions can contribute to better housing outcomes (Stockwell et al., 2013; Bonn et al., 2020; Brocious et al., 2021).

No other significant differences were found between accepters and non-accepters which is surprising as we would think those with favorable healthcare experiences or social support as well as those struggling to access healthcare services, would be more receptive to interventions. We hope that studying qualitative data will help to clarify this subject.

It should be noted that 40% of PEH with self-reported ARP were experiencing homelessness for more than 3 years in this sample. Interestingly, this was not associated with worse intervention acceptance nor with worse appointment adherence. This suggests that treatment efforts can be accepted even by those who have been experiencing homelessness for longer periods, particularly where emergency shelters provide a new setting and opportunity for care. Previous contact with health services did not seem to affect acceptance of harm reduction interventions.

Regarding social data, 38.3% of interviewees had been homeless for < 1 year, the same as 2018 national reports that placed this figure at 38.5%. From December 2019 to December 2020, the number of people experiencing houselessness increased by 27%, and the number of people experiencing rooflessness by only 5%. This may reflect positive policy implements with new structures such as emergency shelters stopping time spent roofless from increasing during the social upheaval of the pandemic (Grupo de Trabalho para a Monitorização e Avaliação da ENIPSSA, 2020, 2021; De Diário, 2022).

Overall, 26% of the sample were not Portuguese citizens, with 44.4% of non-Portuguese citizens in a homeless situation for < 6 months (against 13.7% of patients with Portuguese citizenship) and 50% for < 1 year against 27.4% seen in Portuguese citizens, which is a probable reflection of the pandemics impact on migration challenges and housing crisis, affecting more disproportionally those with more precarious jobs and less social support. Although without statistical evidence, the majority of migrants from Asia and Africa, who self-reported having an ARP, against Portugal-born citizens, adhered to this intervention, which can represent a low-threshold intervention role in bridging medical services in a well-known access gap (Lemmens et al., 2017). Further qualitative data can help to explore these findings.

Our sample included nine women who self-reported having an ARP. The previous experience of women's vulnerability in mixed shelters suggested a need for specific gender interventions. Therefore, a special emphasis was made to admit all women in need, including couples and trans-women (none self-reported as being transgender persons in our sample). Considering the lower prevalence of ARP in women in general, this number may also reflect gender stigma and a more hidden consumption pattern although this is speculative (Braud and Loison-Leruste, 2022).

Limitations to this study include a lack of formal diagnosis or standardized alcoholism classifications as well as a broad definition of homelessness (we could not specify between roofless, houseless, or insecure housing situations although we admit most of our sample to be roofless until shelter).

Research into care for homeless people can be challenging. There are difficulties in maintaining a constant follow-up (due to lack of a fixed address or easy-to-reach contact), often mistrust against carers or interventions, extreme power imbalances between researchers and research subjects, rapidly changing situations, stigma from the medical community, and multiple comorbidities.

Purely quantitative studies may provide a clouded picture, withholding context, perceptions, and motivations which motivated a mixed-methods approach with qualitative data following soon.

## 5. Conclusion

The 2020 COVID-19 pandemic destabilized the already insufficient healthcare and social systems, bringing further hardships upon those with fewer resources. People experiencing homelessness and those with substance use disorders represent an especially fragile subset of the population, often neglected and at risk for health complications, poor healthcare access, and perpetuation of homelessness.

Rapid response strategies such as emergency shelters, quick access medical consults, and low-threshold pharmacological interventions provided immediate relief as well as an opportunity to reframe care and health approaches in this population.

In this sample, 39% adhered to some form of intervention (pharmacological or alcohol-use appointment). Pharmacological intervention adherence reached 36.2% and was associated with appointment adherence and having withdrawal symptoms while being independent of time experiencing homelessness, substance use, and other analyzed variables. Qualitative perspectives from patients, technicians, and other groups should be sought to deepen understanding and inform future works. Reasons for non-adherence must be explored and mitigated to increase engagement. The potential of pharmacological intervention in social settings should be further analyzed as a strategy to increase acceptance and adherence to more structured medical interventions.

## Data availability statement

The raw data supporting the conclusions of this article will be made available by the authors, without undue reservation.

## Ethics statement

The studies involving human participants were reviewed and approved by Comissão de Ética da Associação Regional de Saúde de Lisboa (Regional Health Administration Ethical Commission). The Ethics Committee waived the requirement of written informed consent for participation.

## Author contributions

FA, RM, AN, AVS, CP, JT, RF, and SD contributed to the conception and design of the study. FA organized the database. FA and AS performed the statistical analysis. FA, RM, and AN wrote the first draft of the manuscript. FA, RM, AN, AG, and AS wrote sections of the manuscript. All authors contributed to the manuscript revision, read, and approved the submitted version.
